# Reflex Anuria: A Complication of Hyperthermic Intraperitoneal Chemotherapy

**DOI:** 10.7759/cureus.20269

**Published:** 2021-12-08

**Authors:** Mohamed Fayed, Gargi Banerjee, Danni Feng, Irene Chen

**Affiliations:** 1 Anesthesiology and Perioperative Medicine, Henry Ford Health System, Detroit, USA; 2 Anesthesiology, Pain Management and Perioperative Medicine, Henry Ford Health System, Detroit, USA; 3 Anesthesiology, Wayne State University School of Medicine, Detroit, USA; 4 Anesthesiology, Wayne State University, Detroit, USA

**Keywords:** uro oncology surgery, obstructive uropathy, mild hydronephrosis, icu patients, decrease urine output, ureteric stent, acute renal failure and hemodialysis in icu, acute renal failure, cytoreductive surgery and hipec, reflex anuria

## Abstract

Reflex anuria (RA) is a rare cause of abrupt reduction of urine output following trauma, irritation, or painful stimuli to the kidneys, ureters, or surrounding organs. The mechanism of RA is a reflex spasm of both ureters and/or renal arterioles. It is a well-documented complication of colorectal surgeries and gynecological surgeries which involve placement of a ureteric stent for ureteric identification and prevention of injury. RA and post-renal obstruction can both be complications of intraperitoneal hyperthermic chemotherapy (HIPEC) in patients who are undergoing surgery for colorectal cancer and peritoneal carcinomatosis. HIPEC procedure can lead to inflammation of the entire abdomen, including the ureters. This inflammation can result in hematuria that can form clots along the urinary tract and cause post-renal obstruction. The inflammation can also result in RA. It is essential to maintain high urine output during the early postoperative period to prevent clots and the ensuing post renal obstruction. It is also important to identify RA and maintain a low threshold to treat it by placing ureteric stents even in the absence of overt bilateral hydronephrosis.

## Introduction

Reflex anuria (RA) is a rare cause of acute kidney injury following abdominal and pelvic surgeries secondary to irritation or stimulation of the kidneys, ureters, or surrounding organs [1,2]. The current and widely accepted definition of RA, as proposed by Hull et al. 1980, is the cessation of urine output from both kidneys in response to irritation or trauma to one kidney or its ureter, or in response to severely painful stimuli to surrounding organs [2]. There are two proposed mechanisms. The first one involves a diffuse spasm of the intrarenal arterioles sufficient to significantly decrease glomerular filtration. The second hypothesis postulates the spasm of both ureters resulting in a functional obstruction [3].

RA had been reported in an elective gynecological surgery where a patient underwent myomectomy with preoperative bilateral prophylactic ureteric catheterization for the treatment of two large intramural fibroids. There were no intra-operative complications. Ureteric stents were removed the following day. On the third postoperative day, the patient developed anuria with signs of AKI. Pre- and intrarenal causes were ruled out. Ultrasonography showed bilateral moderate hydronephrosis with an empty bladder. Retrograde pyelography did not show any evidence of obstruction, but a bilateral ureteric stent was still placed. The patient started diuresing immediately after stenting [4]. Another case report shows a case of RA presenting two days after a patient undergoing a bilateral retrograde pyelography for hematuria. The patient’s retrograde pyelography procedure was uncomplicated. No apparent abnormality such as malignancy or urolithiasis could be detected, but pyelorenal extravasation of contrast was remarkable. The diagnosis of RA was made after ruling out all causes of pre and intrarenal acute kidney injury and also post renal obstruction. The condition resolved with one hemodialysis session [5]. RA has also been described as a possible mechanism of acute kidney injury in a case of colorectal surgery which involved the placement of ureteric stents [6]. In another case report, the author did not find cause for acute renal failure (ARF), which resolved with the performance of bilateral nephrectomies. It was labeled as RA due to an unknown cause [7].

So far, RA has not been linked with HIPEC procedures. We are presenting a case of RA that developed after intra-abdominal HIPEC. The patient needed reinsertion of ureteric stents to relieve bilateral ureteric obstructions which resulted from RA. In this case report, we want to emphasize the importance of identifying RA and provide some preventative and treatment options.

## Case presentation

A 74-year-old male patient with a past medical history of well-controlled hypertension, colon adenocarcinoma, and peritoneal carcinomatosis presented to our hospital for cytoreductive surgery, and HIPEC. During surgery, the urology team inserted bilateral ureteral stents for ureteral identification. The patient had bilateral ureterolysis, and widespread peritonectomy followed by HIPEC with Mitomycin C for 90 minutes. Ureteral catheters were removed at the end of the surgery. Intra-operatively, he received a balanced electrolyte solution with a total input of 8 liters of fluid and a urine output of 720 mL. The estimated blood loss was 700 mL. The patient was hemodynamically stable throughout the procedure. His blood pressure ranged from 110/60 to 140/80 mmHg and his heart rate ranged from 80 to 100 beats per minute.

**Figure 1:**
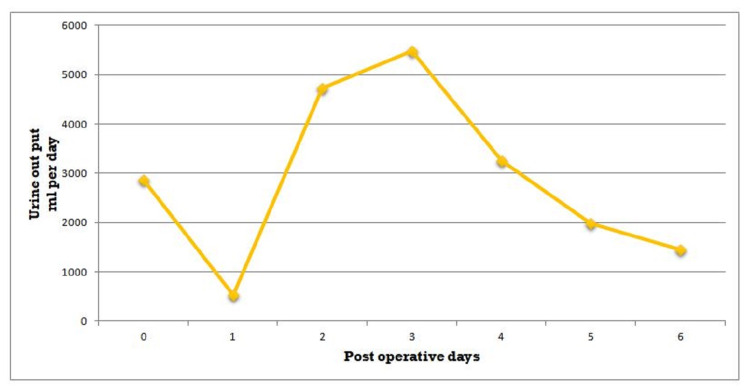
Patient's urine output during the hospital course.

**Figure 2:**
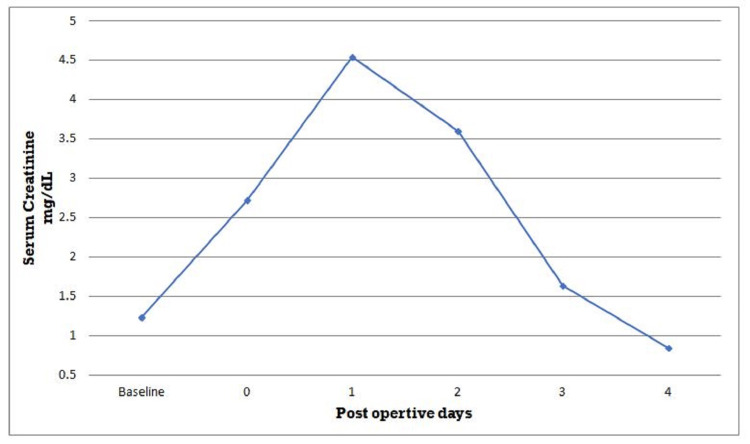
Patient's serum creatinine during the hospital course.

The patient was transferred to the surgical intensive care unit for observation. He made a total of 2,800 cc of urine on an operative day. However, on postoperative day 1, the patient developed hematuria and had low urine output, with total urine output of 500 cc over 24 hours (Figure 1). He received a total of 3 liters of fluid that day including albumin 5%. The patient endorsed abdominal pain and inability to open eyes due to peri-orbital edema. Creatinine acutely increased from 1.2 mg/dL to 4.18 mg/dL over 24 hours (Figure 2). The patient had not received any nephrotoxic agents. To promote diuresis, he was given multiple doses of 5% albumin with increasing doses of furosemide. Investigations were done including renal ultrasound, urine analysis, and casts, as well as trends in renal function were assessed. The renal ultrasound did not show hydronephrosis. It showed a right kidney of 11.5 cm along with dilated pelvis. The left kidney was found to be 10.4 cm.

**Figure 3:**
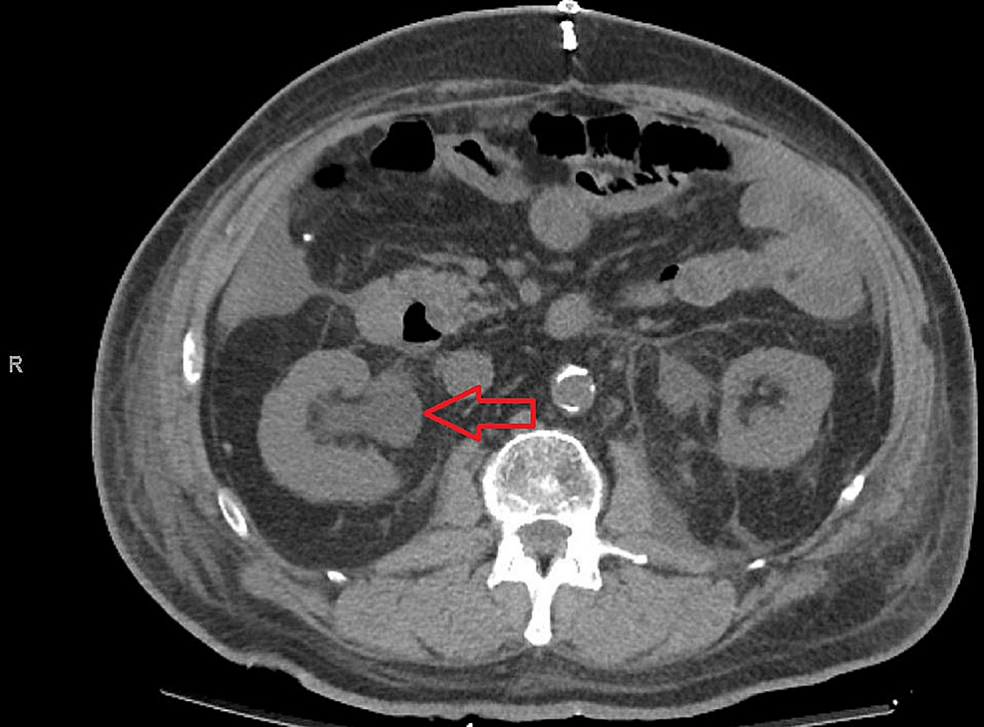
Noncontrast CT scan of the abdomen in our patient. Right renal pelvis showing an increase in size (red arrow).

On postoperative day 2, the patient’s renal functions continued to deteriorate (Figure 2). The creatinine level went up to 4.54 mg/dL. Noncontrast CT scan of the abdomen and pelvis showed mild right-sided hydronephrosis (Figure 3). The right renal pelvic diameter had increased from about 24 to 25 mm compared to his recent renal ultrasound. The left kidney showed mild hydroureter as well. After consultation with the urological team, a decision was made to do cystoscopy and retrograde pyelography. Gross hematuria from bilateral ureteral orifices was noted. On retrograde pyelogram, some ureteric clots were noted but they were not significant, and post-renal obstruction was ruled out. Bilateral ureteric stents were placed in both ureters to relieve any obstruction and also consider RA as a possible cause. Immediately after the placement of ureteric stents, the patient’s urine output started to increase, and he even started having increased urine output (Figure 1). Creatinine began to improve immediately and reached the normal range within a day (Figure 2). Ureteric stents were removed at the bedside on postoperative day 6. He was discharged home on postoperative day 10.

## Discussion

We are presenting the case of a 74-year-old male patient who developed RA secondary to ureteric trauma and associated hematuria. Repeat ureteric stenting was performed. It led to an immediate improvement of his renal function and normalization of his urine output.

RA is rare and it is a diagnosis of exclusion. While it is important to consider common causes of ARF such as prerenal and intrarenal etiology first and manage obvious predisposing conditions like shock and sepsis, it is also important to consider rarer conditions like RA. Our patient developed an abrupt drop in urine output that was associated with deterioration in renal function following abdominal surgery. Pre-renal causes were ruled out, as the patient had stable hemodynamic status, and his means arterial blood pressure was maintained above 70 mmHg. Also, his blood urea nitrogen to creatinine ratio was less than 20, unfortunately, urine analysis was not sent to check on FENA. His hemoglobin was always above 10 gm/dL. It was unlikely to be acute tubular necrosis as there were no signs of ischemia or sepsis and the patient did not receive any nephrotoxic drugs. The urine analysis also did not show any brown, muddy casts. Acute interstitial nephritis was also unlikely since in those cases there is often a slower progression of rising in creatinine than witnessed in our patient. Also, urine analysis was negative for white cell cast. The typical signs and symptoms of post-renal obstruction were missing in this case. Although there was an abrupt decline in renal function, there was an absence of progressive dilatation of the ureters and kidneys. There was no flank pain radiating to the scrotum or groin. Clots were noticed but they were not significant to cause a bilateral renal shutdown. RA was suspected when all other possible causes were ruled out. Also, the treatment for post-renal obstruction and RA is the same: ureteric stent placement along with supportive care.

While it is reported that HIPEC is associated with an increase in the incidence of AKI, there is a lack of information on the association and possible causation of RA by HIPEC [8]. HIPEC procedure causes inflammation of the whole abdomen, including the ureters. This inflammation can result in hematuria. Clots can form along the urinary tract. Both post-renal obstruction and RA are possible complications and they both may present with oliguria or anuria. However, RA causes bilateral intra-renal arteriolar spasm or ureteric spasm and presents without significant hydronephrosis. 

A way to prevent these complications is screening patients who have had prior urinary tract disease or trauma. Adequate hydration prior to the HIPEC procedure may also help with the target urine output being more than 2 mL/kg/hr during the early postoperative course. We further recommend that ureteric stents when placed for ureteric identification during surgical procedures like gynecological, colorectal, or HIPEC procedures, be left in place for the immediate postoperative period and removed in one or two days. They may help to prevent both post-renal obstructions and may mitigate RA. Should oliguria and AKI set in the postoperative setting of HIPEC, without significant hydronephrosis, RA should be considered a differential and a ureteric stent may be placed for treatment.

## Conclusions

RA is a potential complication of the HIPEC procedure. HIPEC procedure can lead to inflammation of the entire abdomen including the ureters that can lead to post-renal obstruction as a result of blood clots in the ureters, bladder, or urethra. It can also cause RA, which presents similarly but without evidence of significant bilateral obstruction. It is important to diagnose RA as it can progress to AKI necessitating renal replacement therapy.
